# Comparative Evaluation of Feature Selection Algorithms for Predicting Radiation-Induced Complications in Head and Neck Cancer Using Multimodal CT and MRI Radiomics

**DOI:** 10.3390/cancers18193200

**Published:** 2026-10-03

**Authors:** Benyamin Khajetash, Mohammad Reza Deevband, William A. Stokes, Meysam Tavakoli

**Affiliations:** 1 Department of Medical Physics, School of Medicine, Iran University of Medical Sciences, Tehran 1449614535, Iran; 2 Department of Biomedical Engineering and Medical Physics, School of Medicine, Shahid Beheshti University of Medical Sciences, Tehran 1985717443, Iran; 3 Department of Radiation Oncology, Winship Cancer Institute, Emory University, Atlanta, GA 30322, USA; 4 Department of Radiation Oncology, Northside Hospital, Atlanta, GA 30342, USA

**Keywords:** radiomics, machine learning, head and neck cancer, feature selection, CT, MRI

## Abstract

Head and neck cancer (HNC) presents a significant challenge due to its anatomic complexity and treatment-related complications such as sticky saliva and xerostomia, which critically impair patients’ quality of life. Recent advancements in imaging technologies, using the concept of radiomics, offer promising approaches for predicting these complications and ultimately reducing their incidence. In this regard, robust feature selection is critical in radiomics-based predictive modeling due to the high dimensionality, redundancy, and instability of extracted imaging biomarkers. This study aims to evaluate nine distinct feature selection algorithms—Boruta, Kruskal, Recursive Maximum Relevance minimum redundancy (R-MRmr), Relief, Recursive Feature Elimination (RFE), Forward Selection (FS), Backward Elimination (BE), Least Absolute Shrinkage and Selection Operator (LASSO), and Principal Component Analysis (PCA)—as a feature-transformation comparator for predicting sticky saliva and xerostomia in HNC patients using clinical, dosimetric, MRI and CT radiomics data. The study involved 250 HNC patients who underwent radiotherapy from three different institutions. From MRI and CT images, a total of 321 radiomic features (107 from each CT, $T_{1}$, and $T_{2}$-weighted MRI) along with 20 clinical and dosimetric features were extracted. The predictive robustness of feature selection methods was further evaluated across five machine-learning classifiers, including K-nearest neighbors (KNN), support vector machine (SVM), random forest (RF), logistic regression (LR), and eXtreme Gradient Boosting (XGBoost), were initially benchmarked using a common feature selection procedure, after which SVM was selected as the reference classifier for systematic comparison of the feature-reduction approaches. Model performance was assessed using metrics objectives such as Area Under the Curve (AUC), accuracy, sensitivity, and specificity. Among the evaluated feature selection approaches, R-MRmr and Relief generally yielded better discrimination, although performance varied according to toxicity endpoint and imaging modality. The highest AUCs were 0.85 for early sticky saliva (R-MRmr, $T_{1}$-weighted MRI), 0.96 for early xerostomia (R-MRmr, $T_{1}$-weighted MRI), 0.65 for late sticky saliva (Relief, combined CT+$T_{1}$+$T_{2}$), and 0.88 for late xerostomia (Relief, $T_{1}$-weighted MRI). $T_{1}$-weighted MRI frequently provided better individual-modality performance, whereas combining all three imaging modalities did not consistently improve discrimination. These findings demonstrate that the choice of feature selection strategy and imaging modality can materially influence radiomics-based toxicity prediction.

## Simple Summary

Radiotherapy for head and neck cancer can cause xerostomia and sticky saliva, which can substantially affect patients’ quality of life. Radiomics uses quantitative information extracted from medical images to help predict these complications. However, radiomics produces many features, and selecting the most informative ones is an important step in developing predictive models. In this study, we compared nine feature selection approaches using CT and MRI radiomics, clinical information, and radiation dose data from 250 patients treated at three institutions. We evaluated predictions of early and late xerostomia and sticky saliva. Among the different methods, Recursive Maximum Relevance minimum redundancy and Relief generally provided favorable results, particularly when combined with $T_{1}$-weighted MRI. These findings highlight the importance of selecting appropriate feature selection strategies when developing radiomics-based models for radiation-induced toxicity. Further validation is needed before these models can be used in clinical practice.

## 1. Introduction

Head and neck cancer (HNC) presents a formidable challenge in oncology due to its complex and heterogeneous nature, encompassing a broad range of etiological factors and clinical manifestations [1]. Radiotherapy plays an integral role in the management of HNC but comes with some considerable attendant toxicity [2,3]. Among the various complications associated with HNC, particularly those resulting from radiotherapy, sticky saliva and xerostomia are especially debilitating [4]. These conditions significantly impair patients’ quality of life, affecting their ability to eat, speak, and maintain oral hygiene, which in turn impacts overall treatment outcomes and long-term recovery [5,6,7].

In radiation oncology, rapid-learning systems are gaining traction, utilizing routine clinical data to develop predictive models for patient-specific treatment outcomes. Beyond outcome prediction, the objective of these decision support systems is to enhance overall patient care and guide the personalization of treatment plans for individual patients [8,9]. Decades ago normal tissue complication probability (NTCP) models emerged for predicting treatment toxicity based on a mathematical function incorporating various input variables such as clinical, and demographic factors, as well as baseline symptoms [10]. These models, derived from the Lyman–Kutcher–Burman model [11], have limited learning capacity because they rely on user feedback to establish the correct correlations between inputs and predictions, making the process largely empirical. Moreover, there remains unexplained variability in NTCP model predictions [12], which could potentially be reduced by incorporating dose-independent radiomic-based approaches, enabling the early identification of high-risk patients before treatment planning [13].

In the same direction, recent advancements in imaging technologies, notably magnetic resonance imaging (MRI) and computed tomography (CT), have revolutionized the way we assess and manage HNC [14]. These modalities, when added to predictive models have huge impact in identifying of various treatment-related outcomes, including structural alterations, toxicities, doses to organs-at-risk, complications, treatment failure, oncological endpoints, and pathological observations [15]. In fact, applying predictive models helps to identify treatment-related toxicities in different cancers and support the personalization of treatment plans. These models require the integration of multi-variable approaches that process diverse data sources, such as clinical records, dosimetric parameters, and a variety of quantitative data derived from different imaging modalities, known as radiomics features [16,17].

Radiomics offers a promising approach for predicting and monitoring treatment-related complications. It involves the extraction of a large number of quantitative features from medical images, which can provide detailed insights into tumor characteristics, treatment responses, and potential complications [18,19]. Moreover, the combination of radiomics and machine learning (ML) models creates strong tools for predicting treatment outcomes that overcome the drawbacks of NTCP models [13,20]. The ML-based radiomics models have been applied to a variety of radiotherapy aspects such as outcome and toxicity prediction [21,22], tumor response modelling [23], treatment planning [24,25], and target delineation [26]. In HNC, radiomics has demonstrated prognostic value and have been validated across multiple institutions [27,28,29,30].

In the context of radiomics, one of the important steps is using appropriate feature selection techniques to identify the most relevant imaging biomarkers associated with specific clinical outcomes. Effective feature selection is crucial not only for enhancing the predictive performance of radiomics-based models but also for improving their interpretability and generalizability [31,32]. Various techniques, such as Boruta, Kruskal, Recursive Max-Relevance Min-Redundancy (R-MRmr), Relief, and Recursive Feature Elimination (RFE), each have their strengths and limitations in filtering out the most significant features from a large dataset [33,34]. Understanding which feature selection technique provides the most accurate and reliable predictions for HNC complications can significantly influence personalized treatment planning and early intervention strategies. This knowledge is essential for developing robust predictive models and decision support tools that can enhance clinical practice and patient outcomes [35,36].

Although previous studies have explored the role of radiomics [16,37,38,39,40], harmonization techniques [19,41,42,43,44], ensemble learning [45,46,47], and classifier comparisons [48,49] in predicting radiation-induced toxicities in HNC, the impact of feature selection strategies on model robustness and generalizability remains insufficiently investigated. In high-dimensional radiomics analysis, different feature selection algorithms may identify substantially different imaging biomarkers due to variations in redundancy reduction, relevance estimation, and statistical dependency assumptions. Consequently, predictive performance, reproducibility, and model stability may vary considerably depending on the selected feature selection approach. Therefore, a systematic benchmarking of feature selection algorithms is essential to establish reliable and clinically translatable radiomics-based toxicity prediction models.

This study aims to systematically evaluate nine feature-reduction approaches for predicting radiation-induced sticky saliva and xerostomia in HNC patients using CT and MRI radiomics, clinical, and dosimetric data. The evaluated approaches comprise eight feature selection methods—Boruta, Kruskal–Wallis, R-MRmr, Relief, RFE, Forward Selection (FS), Backward Elimination (BE), and LASSO—and PCA as a feature-transformation and dimensionality-reduction comparator. By comparing these approaches across different imaging configurations and toxicity endpoints, we aim to investigate how the choice of feature-reduction strategy influences predictive performance and to identify potentially informative approaches for future radiomics-based toxicity prediction studies.

## 2. Materials and Methods

**Figure 1:**
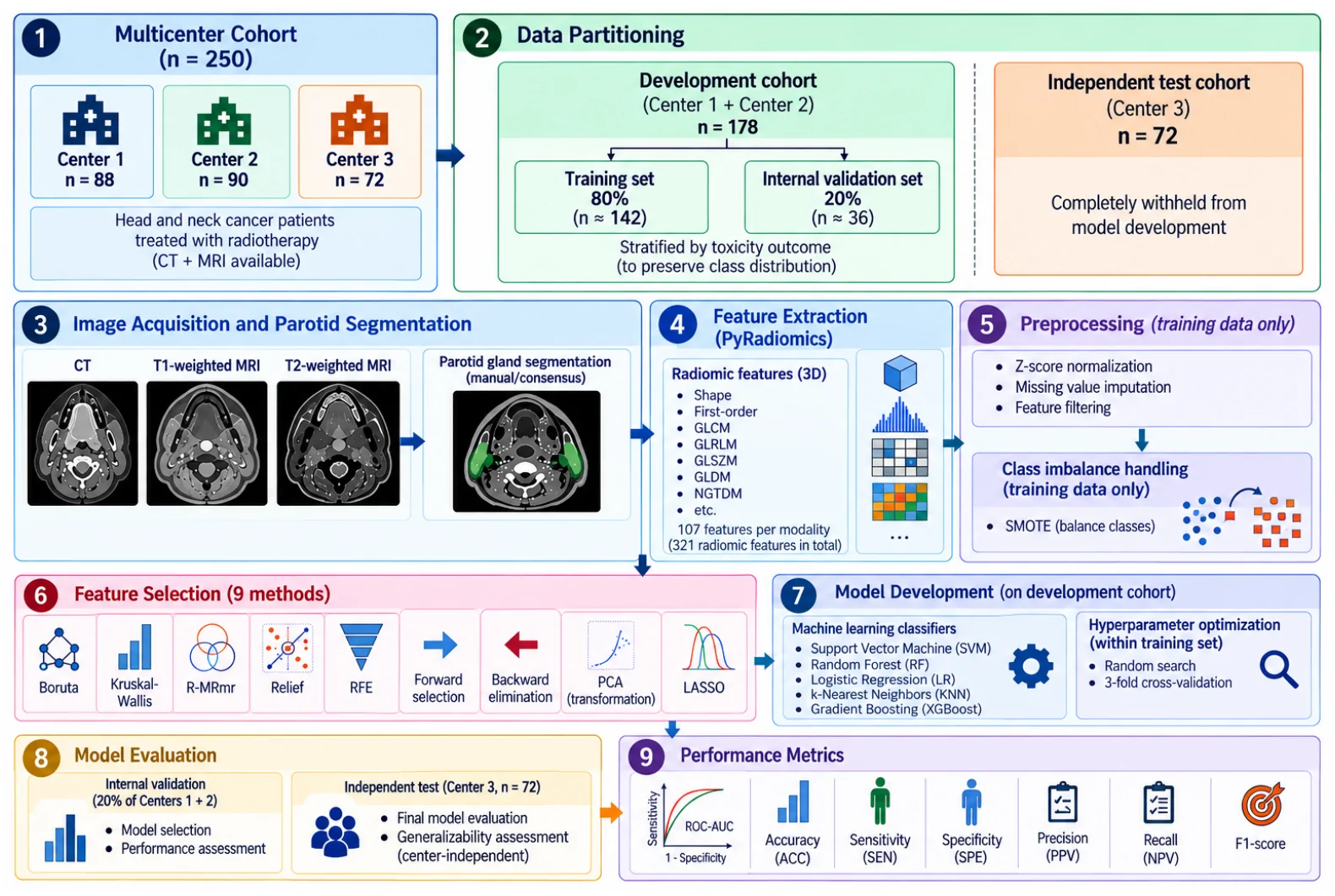
Overview of the study design and radiomics-based ML workflow. The multicenter cohort included 250 patients from three institutions. Patients from Centers 1 and 2 (n = 178) constituted the development cohort and were divided into 80% training and 20% internal validation subsets, whereas Center 3 (n = 72) was completely withheld from model development and used as an independent institutional test cohort. The held-out 20% subset was used for internal validation, whereas the independent Center 3 cohort remained untouched until final model evaluation.

The primary purpose of this work was to predict both sticky saliva and xerostomia after radiotherapy in HNC patients. As schematically illustrated in Figure 1, the workflow was developed in different steps. The study began by acquiring MRI and CT images from patients and defining regions of interest. We combined imaging-derived radiomics features with clinical and dosimetric variables and evaluated nine feature selection approaches to identify informative representations for subsequent predictive modeling. A Support Vector Machine (SVM) classifier was used to build the predictive model.

### 2.1. Patient Selection

A prospective evaluation was conducted involving 250 patients with HNC to assess the side effects at different stages of treatment. The study included patients who received radiation therapy between 2020 and 2022, with the parotid gland being identified as a critical organ at risk. Patients with treatment interruptions or dental implants were excluded due to potential artifact risks. The final multicenter cohort consisted of 250 patients recruited from three participating institutions. Centers 1, 2, and 3 contributed 88, 90, and 72 patients, respectively. For model development and validation, patients from Centers 1 and 2 (n = 178) constituted the development cohort, whereas all patients from Center 3 (n = 72) were reserved as an independent center-specific test cohort. Detailed imaging acquisition parameters for the participating centers and demographic, clinical, treatment, and outcome characteristics of the study population are provided in Tables S1 and S2 of the Supplementary Materials.

The primary treatment modality was Intensity-Modulated Radiation Therapy (IMRT), used for conditions including nasopharyngeal, hypopharyngeal, oropharyngeal cancers, and lymphoma. Among the cohort, 71% experienced early xerostomia (EarlyX) and 44% had late xerostomia (LateX). Additionally, 55% of patients exhibited early symptoms of sticky saliva (EarlySS), while 16% had late symptoms (LateSS).

### 2.2. Image Acquisition and Treatment Planning

Before starting radiotherapy, CT and MRI were taken, which were essential for delineating the Gross Tumor Volume (GTV) and organs at risk (OARs). Treatment planning was conducted using the Eclipse system version 15 (Varian Medical Systems, Palo Alto, CA, USA). CT images were acquired with a GE Hi-Speed CT scanner (GE Healthcare, Milwaukee, WI, USA) set to a slice thickness of 1.5 mm, a field of view of 500 mm, a matrix size of 512×512, a tube voltage of 120 kV, and a tube current of 340 mAs. The specific MRI protocols used at three different centers are detailed in Table S1 in the Supplementary Materials.

Radiation-induced xerostomia and sticky saliva were assessed using the European Organisation for Research and Treatment of Cancer Quality of Life Questionnaire–Head and Neck 35 module (EORTC QLQ-H&N35) [50]. Xerostomia was assessed using the dry-mouth item (HNDR), whereas sticky saliva was assessed using the corresponding sticky-saliva item (HNSS), representing separate patient-reported symptom endpoints. Responses were recorded on a four-point scale ranging from 1 (“not at all”) to 4 (“very much”), with higher responses indicating greater symptom burden. For the present classification analysis, responses were dichotomized to distinguish moderate-to-severe symptoms from absent or mild symptoms: responses of 1 or 2 were classified as absence of toxicity, whereas responses of 3 or 4 were classified as presence of toxicity. Early toxicity was defined using the EORTC QLQ-H&N35 assessment obtained three months after completion of radiotherapy, whereas late toxicity was defined using the assessment obtained six months after completion of radiotherapy. Accordingly, four binary patient-reported endpoints were analyzed independently: EarlyX, EarlySS, LateX, and LateSS.

### 2.3. Feature Extraction

Prior to radiomic feature extraction, CT and MR images were registered using the Eclipse treatment planning system. The bilateral parotid glands were independently delineated on the registered CT and MR images by two experienced radiation oncologists. The contours were subsequently reviewed by both radiation oncologists, and any discrepancies were resolved by consensus to establish the final parotid contours used for radiomics feature extraction.

To reduce inter-institutional variability associated with differences in scanner hardware and imaging acquisition protocols, radiomics features were harmonized across centers using the ComBat harmonization approach, consistent with our previously described methodology [43,51]. Harmonization was performed to mitigate center- and acquisition-related effects while preserving biologically relevant interpatient variability. Radiomics features were extracted from the segmented parotid glands using PyRadiomics (version 3) [52] according to standardized radiomic feature definitions. Prior to feature extraction, CT and MR images were resampled to an isotropic voxel spacing of 1 × 1 × 1 mm^3^ using B-spline interpolation to standardize voxel dimensions across imaging datasets. Image intensities were discretized using a fixed number of 32 gray-level bins. Radiomics features were calculated in three dimensions from the original images without additional filtering. Seven feature classes were extracted, including shape, first-order statistics, gray-level co-occurrence matrix (GLCM), gray-level run-length matrix (GLRLM), gray-level size-zone matrix (GLSZM), gray-level dependence matrix (GLDM), and neighboring gray-tone difference matrix (NGTDM). A total of 107 radiomic features were extracted from each imaging modality (CT, $T_{1}$, and $T_{2}$-weighted MRI), resulting in 321 imaging features before feature reduction. The 107 features comprised 13 shape, 19 first-order, 24 GLCM, 16 GLRLM, 16 GLSZM, 14 GLDM, and 5 NGTDM features.

Because radiomics datasets typically contain highly correlated and redundant variables, feature selection was considered a critical step to minimize overfitting, improve model interpretability, and enhance predictive reproducibility. Radiomics extraction parameters were kept consistent within each imaging modality. The extraction settings included the PyRadiomics version, voxel resampling strategy and target spacing, interpolation method, intensity normalization, gray-level discretization/bin width, and enabled image and feature classes.

For all patients, clinical features extracted included the age, sex, family history of cancer, chemotherapy, surgery, smoking status. In addition, Dose-Volume Histogram (DVH) analysis was used to determine the volumes of the parotid glands receiving doses between 5 Gy ($V_{5Gy}$) to 70 Gy ($V_{70Gy}$). Additional treatment planning data, including parotid volume and various dose metrics, were collected. A total of 20 DVH-related and and clinical features were derived from this analysis. Tables S2 and S3 in the Supplementary Materials presented the details of all dosimetric and clinical features.

### 2.4. Feature Selection

In order to investigate the effect of feature selection algorithms on the performance of the final model to predict the sticky saliva and xerostomia complications, nine different algorithms were used. These algorithms included Boruta, Kruskal, R-MRmr, Relief, RFE, FS, BE, PCA, and LASSO.

The evaluated methods represent different approaches to feature selection. Wrapper-based methods [53], including FS, BE, Boruta, and RFE, select subsets of variables through an iterative procedure in which feature inclusion or removal is guided by a model-dependent criterion. Boruta is a Random Forest-based wrapper method that compares the importance of original variables with randomly permuted shadow features to identify relevant predictors [54]. RFE recursively removes the least informative variables according to model-derived feature importance until an optimal subset is obtained [55]. Filter-based methods [53], including the Relief, and R-MRmr, evaluate features using statistical or data-driven relevance criteria that are independent of the final predictive classifier. Relief assigns feature weights according to the ability of individual variables to differentiate neighboring observations belonging to different outcome classes. R-MRmr aims to identify informative variables by maximizing their relevance to the clinical endpoint while simultaneously minimizing redundancy among the selected features [56]. LASSO represents an embedded feature selection approach [57], in which variable selection occurs during model fitting through an L1 regularization penalty that shrinks the coefficients of less informative predictors toward zero. PCA differs conceptually from the other methods because it is a feature transformation rather than a direct feature selection technique. PCA projects the original correlated variables onto a smaller set of orthogonal principal components that preserve a substantial proportion of the variance in the original feature space. PCA was therefore included as a dimensionality-reduction comparator rather than as a conventional feature selection algorithm [58]. Implementation parameters for the nine evaluated feature selection approaches are summarized in Table S13.

### 2.5. Machine Learning Modeling

Predictive performance was evaluated using four feature-set configurations: (1) CT, (2) $T_{1}$-weighted MRI, (3) $T_{2}$-weighted MRI, and (4) CT + $T_{1}$ + $T_{2}$-weighted MRI. In these categories, we also added all clinical and dsimetric features. Patients from Centers 1 and 2 (n = 178) were combined to form the development cohort and were divided into training (80%) and internal validation (20%) subsets. The 80% training subset was used for model development, including repeated three-fold cross-validation for model training and hyperparameter optimization, whereas the 20% internal validation subset was held out from the cross-validation procedure and used for internal evaluation of the developed models. All patients from Center 3 (n = 72) were completely withheld from model development and constituted an independent institutional test cohort. Following completion of model development and internal validation, the selected models were evaluated on the untouched Center 3 cohort to assess inter-institutional generalizability.

The repeated model-development procedure generated multiple fitted models, each of which was subsequently evaluated on the complete, unchanged Center 3 test cohort. Center 3 was used exclusively for independent testing and did not participate in model training, feature selection, estimation of normalization parameters, SMOTE, or hyperparameter optimization. Performance metrics were calculated separately on Center 3 for each fitted model and are reported as the mean ± standard deviation (SD) across these repeated independent-test evaluations. Accordingly, the reported SD reflects variability in test performance across models generated during repeated model development and does not represent cross-validation performed within Center 3.

To prevent information leakage, all data-dependent preprocessing and model-development procedures were restricted to the training data. Within the repeated three-fold cross-validation procedure, z-score normalization parameters were estimated using only the training portion of each cross-validation fold and were subsequently applied unchanged to the corresponding validation fold. Feature selection was likewise performed independently within the training portion of each fold. After model development, the resulting preprocessing transformations, selected feature representation, and optimized model parameters were applied without refitting to the held-out 20% internal validation subset and subsequently to the independent Center 3 test cohort.

To select a reference classifier for the subsequent feature selection comparison, five ML classifiers, including support vector machine (SVM), logistic regression (LR), k-nearest neighbors (KNN), eXtreme Gradient Boosting (XGBoost), and random forest (RF), were initially evaluated. To avoid unnecessarily expanding the benchmarking analysis across all imaging configurations, classifier comparison was restricted to CT and $T_{1}$-weighted MRI, which represented the two imaging modalities demonstrating the strongest overall predictive performance in the preliminary analysis. Classifier performance was evaluated using AUC, accuracy, sensitivity, and specificity. Based on its consistently favorable overall performance across the evaluated outcomes and the two selected imaging modalities, SVM was selected as the reference classifier for the subsequent systematic comparison of feature selection methods. Detailed classifier benchmarking results are provided in Tables S4–S7. For the classifier benchmarking analysis, Relief feature selection algorithm was applied consistently across all classifiers to ensure that classifier performance was compared using an identical feature selection procedure.

The hyperparameters of the evaluated classifiers were optimized within the training data with the area under the receiver operating characteristic curve (ROC-AUC) used as the optimization criterion. The principal hyperparameters considered included the regularization parameter and kernel settings for SVM, regularization strength for LR, number of neighbors and distance weighting for KNN, tree number and depth for RF, and tree depth, learning rate, number of estimators, and sampling parameters for XGBoost. The hyperparameter search space and selected model settings are summarized in Supplementary Materials Table S8.

### 2.6. Statistical Analysis

Model performance was evaluated using the ROC-AUC, accuracy, sensitivity, and specificity. During model development, repeated three-fold cross-validation was performed exclusively within the 80% training subset of the combined Centers 1 and 2 development cohort to support model training and hyperparameter optimization. The held-out 20% internal validation subset was not included in the cross-validation procedure and was used for internal evaluation following model development. The fitted models generated during repeated model development were subsequently applied independently to the complete Center 3 test cohort (n = 72), which remained untouched throughout model development and internal validation. Performance metrics on Center 3 were calculated for each fitted model and are reported as mean ± SD across the repeated independent-test evaluations. Pairwise comparisons of ROC-AUCs between feature-reduction approaches were performed on paired patient-level prediction scores from the same Center 3 subjects using the DeLong test [59,60] for correlated ROC curves. All statistical tests were two-sided. Pairwise DeLong comparisons were exploratory, and the reported *p*-values were not adjusted for multiple comparisons. A nominal threshold of *p* < 0.05 was used to describe the results; these comparisons should not be interpreted as confirmatory evidence of differences between feature selection approaches.

## 3. Results

The main objective of this study was to compare different feature selection algorithms for predicting sticky saliva and xerostomia. Prior to the systematic comparison of feature selection methods, five ML classifiers (SVM, LR, KNN, XGBoost, and RF) were benchmarked using CT- and MRI-based feature sets [49]. These two modalities were selected for classifier benchmarking because they demonstrated better overall predictive performance among the individual imaging modalities evaluated in the preliminary analysis. As summarized in Tables S4–S7, classifier performance varied according to imaging modality and toxicity endpoint; however, SVM demonstrated the most consistently favorable overall performance across the evaluated scenarios and was therefore selected as the reference classifier for the subsequent feature selection analysis.

**Figure 2:**
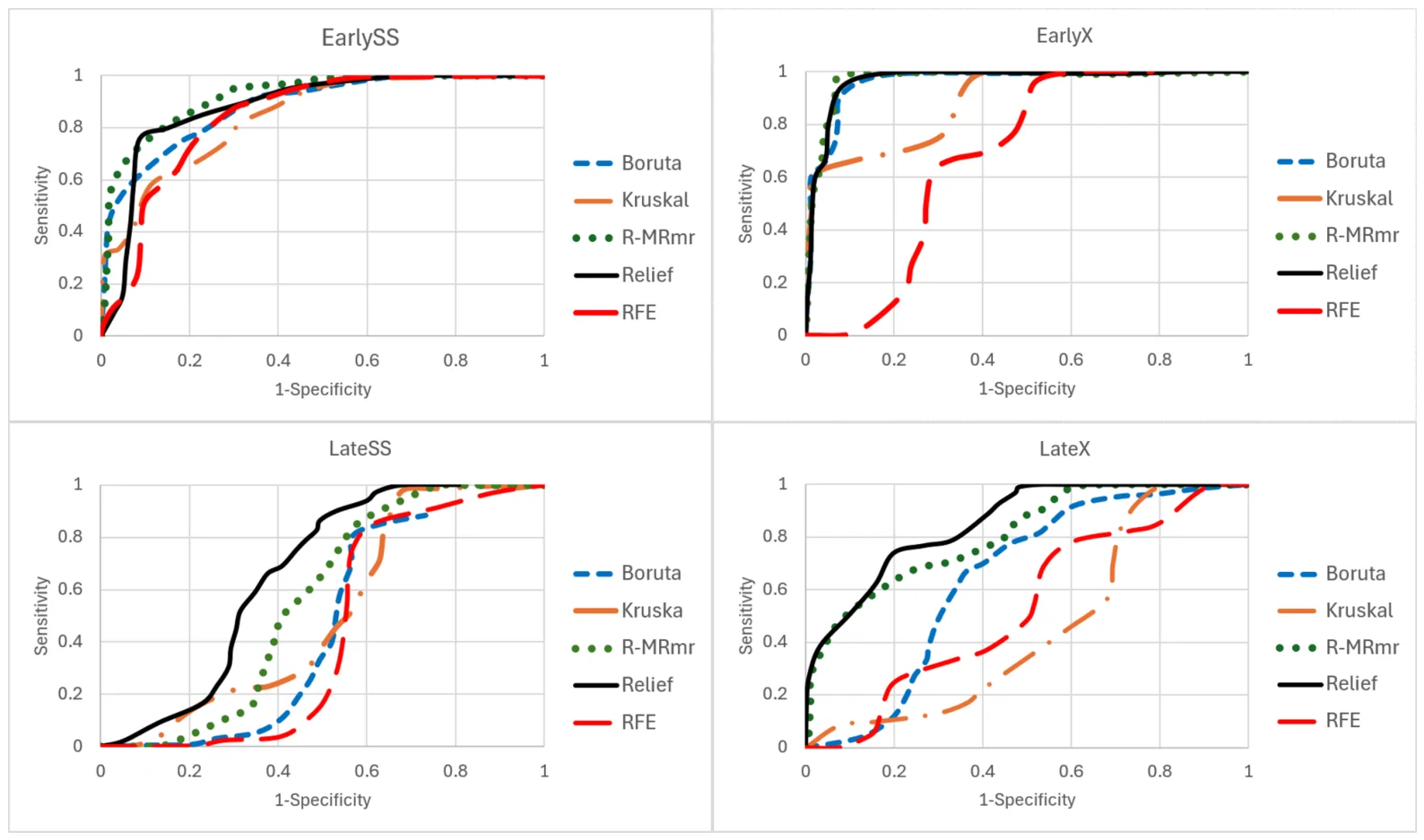
ROC diagrams corresponding to the best performance of different feature selection algorithms (Boruta, Kruskal, R-MRmr, Relief, and RFE) to prediction of both early (**top** panels) and late (**below** panels) of sticky saliva and xerostomia.

In all comparison scenarios we used combination of three different sets of features that we have mentioned above which were clinical, dosimetric, and imaging. At the end, the results of top five ranked feature selection algorithms were presented, and the detailed results of the rest presented in the Supplementary Materials. Figure 2 shows the ROC diagrams related to the best models in predicting early and late xerostomia and sticky saliva.

The findings in this study are reported under four major groups based on the grade of the toxicity including, early and late stage of both sticky saliva and xerostomia.

### 3.1. Group1: Early Sticky Saliva

Based on the patient information data, Section 2.1, among the cohort, 55% of patients exhibited early symptoms of sticky saliva (EarlySS). Table 1 presents a comprehensive comparison of different feature selection algorithms employed with a SVM classifier to predict EarlySS. It outlines the performance metrics, including AUC, ACC, Sensitivity (SEN), and Specificity (SPE), for various combinations of input features such as clinical, dosimetric (DVH), and different imaging modalities (CT, $T_{1}$ and $T_{2}$ MRI). For EarlySS prediction, all the models exhibited promising performance. Among the models evaluated, the highest AUC of 0.85 is achieved using the R-MRmr feature selection algorithm with a combination of clinical, dosimetric, and $T_{1}$-weighted MRI features. Pairwise DeLong testing showed that the AUC obtained with R-MRmr using $T_{1}$-weighted MRI was significantly higher than those obtained with Boruta (*p* = 0.032), Kruskal (*p* = 0.018), and RFE (*p* = 0.041), whereas the difference compared with Relief was not statistically significant (*p* = 0.087).

### 3.2. Group2: Early Xerostomia

From the patients information, 71% experienced early xerostomia (EarlyX). For the EarlyX prediction, Table 2 provides an insightful comparison of various feature selection algorithms. Here, although all the feature selection algorithms performed acceptably in final prediction, R-MRmr, Relief, and Boruta had an AUC of more than 0.90, and achieved the best-performing algorithms with high accuracy and sensitivity, and specificity as well. For EarlyX prediction, the strongest performance was achieved by R-MRmr using clinical, dosimetric, and $T_{1}$-weighted MRI features, with an AUC of 0.96 ± 0.04, accuracy of 0.90 ± 0.10, sensitivity of 0.87 ± 0.10, and specificity of 0.93 ± 0.07. Relief and Boruta using $T_{1}$-weighted MRI also demonstrated high discrimination, with AUCs of 0.92 ± 0.04 and 0.91 ± 0.09, respectively. Pairwise DeLong testing indicated that the AUC obtained with R-MRmr was significantly higher than those obtained with Boruta (*p* = 0.021) and Kruskal (*p* = 0.014), whereas the differences compared with Relief (*p* = 0.073) and RFE (*p* = 0.058) did not reach statistical significance.

### 3.3. Group3: Late Sticky Saliva

Among all the patients, 16% of them exhibited late sticky saliva (LateSS). In predicting LateSS, Table 3 shows a comparative analysis of different feature selection algorithms. The top-performing model in this table, achieved using Relief with Clinical + Dosimetric + CT + $T_{1}$ + $T_{2}$-weighted MRI features, exhibits an AUC of 0.65. Our prediction results indicate the challenges in accurately predicting LateSS cases, highlighting the need for further investigation and refinement of predictive models for this condition. Although Relief with the combined CT + $T_{1}$ + $T_{2}$ feature configuration yielded the highest numerical AUC for LateSS (0.65 ± 0.13), pairwise DeLong testing did not demonstrate statistically significant differences compared with Boruta (*p* = 0.118), Kruskal (*p* = 0.094), R-MRmr (*p* = 0.156), or RFE (*p* = 0.127).

### 3.4. Group4: Late Xerostomia

In predicting late xerostomia (LateX), we have 44% who had LateX among all the cases. Table 4 provides a comparison of different feature selection algorithms. The top-performing model in this table, achieved using Relief with Clinical + Dosimetric + $T_{1}$-weighted MRI features, exhibits an AUC of 0.88, and achieved the best-performing algorithms with high accuracy and sensitivity, and specificity as well. Pairwise DeLong testing showed that the AUC obtained with Relief using T1-weighted MRI was significantly higher than those obtained with Kruskal (*p* = 0.026) and RFE (*p* = 0.039), whereas the differences compared with Boruta (*p* = 0.064) and R-MRmr (*p* = 0.081) were not statistically significant.

Across the four toxicity endpoints, $T_{1}$-weighted MRI frequently demonstrated the strongest individual-modality performance. Combining CT, $T_{1}$, and $T_{2}$-weighted MRI did not consistently improve discrimination compared with the best-performing individual modality, although the combined CT+$T_{1}$+$T_{2}$ configuration yielded the highest AUC for LateSS.

### 3.5. Overall Comparison of the Nine Feature Selection Approaches

To provide a comprehensive comparison of all nine evaluated feature-reduction approaches, Table 5 summarizes the highest AUC achieved by each method across the four imaging configurations for each toxicity endpoint. The relative performance of the methods varied according to the toxicity endpoint. R-MRmr achieved the highest AUC for EarlySS (0.85 ± 0.10) and EarlyX (0.96 ± 0.04), whereas Relief achieved the highest AUC for LateSS (0.65 ± 0.13) and LateX (0.88 ± 0.11). Among the four additional approaches reported in the Supplementary Materials, FS achieved the highest AUC for EarlySS (0.65 ± 0.10) and EarlyX (0.69 ± 0.13), whereas BE achieved the highest AUC for LateX (0.56 ± 0.14). Performance of these additional approaches was generally lower for LateSS, with maximum AUCs ranging from 0.38 to 0.42. Complete modality-specific results are provided in Table 1, Table 2, Table 3 and Table 4 and Supplementary Tables S9–S12.

## 4. Discussion

In this study, we introduced a more comprehensive work to investigate the influence of different feature selection algorithms and radiomics features in the prediction of radiotherapy-induced complications in HNC patients. Another impact of this project is that the common side effects, xerostomia, and sticky saliva, have not been studied simultaneously using CT, $T_{1}$ and $T_{2}$-weighted MRI images, and clinical and dosimetric data in the early and late stages using different feature selection models.

Overall, using the results presented in all tables, we can conclude that among all feature selection algorithms, both R-MRmr and Relief using the $T_{1}$ MRI dataset provided the best results compared to other algorithms. As presented in Table 1, across various input data sets, besides both R-MRmr and Relief, Boruta outperformed well for predicting early sticky saliva. Boruta achieved its best performance when utilizing clinical data with DVH and $T_{1}$ MRI features, yielding an AUC of 0.80 and an accuracy of 0.77. Notably, the inclusion of $T_{2}$ MRI data resulted in lower performance across all feature selection algorithms, with acceptable AUC values for all algorithms between 0.61 to 0.68, indicating the limited relevance of $T_{2}$ MRI in early sticky saliva prediction compared to CT and $T_{1}$ MRI. The early xerostomia, as it can be seen in Table 2, all algorithms except RFE in $T_{1}$ MRI data had high performance in predicting early xerostomia. Again, besides both R-MRmr and Relief, which achieved their best performance when using clinical data alongside DVH and $T_{1}$ MRI features, Boruta achieved high results as well. Here, the high sensitivity indicating the ability to correctly identify the cases of early xerostomia. The inclusion of $T_{2}$ MRI data generally resulted in reduced performance across all the algorithms, emphasizing the limited relevance of $T_{2}$ MRI in early xerostomia prediction compared to CT and $T_{1}$ MRI.

In predicting late sticky saliva (Table 3), all feature selection algorithms showcased varying performance outcomes across input data sets. Using clinical data combined with DVH $T_{1}$, and $T_{2}$ MRI features, yielding a higher AUC and accuracy for both R-MRmr and Relief. However, sensitivity and specificity were not high, indicating challenges in correctly identifying late sticky saliva cases. Relief exhibited relatively better performance with the same input features (AUC of 0.65). Nonetheless, the overall performance remained suboptimal across all algorithms, with limited sensitivity and specificity levels indicating difficulties in accurately identifying late sticky saliva. The late xerostomia prediction was presented across feature selection algorithms and input data sets (Table 4). Utilizing clinical data combined with DVH and $T_{1}$ MRI features, Relief exhibited the highest performance in certain configurations. The inclusion of $T_{2}$ MRI and CT data consistently led to diminished performance across the algorithms, reinforcing the limited utility in late xerostomia prediction compared to $T_{1}$ MRI.

The favorable performance of R-MRmr may be explained by its explicit balance between maximizing relevance to the outcome and minimizing redundancy among candidate features, an important advantage in radiomics datasets where texture variables are often highly correlated. Relief, in contrast, estimates feature importance according to the ability of variables to discriminate neighboring instances from different outcome classes, which may allow it to capture complex local relationships within heterogeneous imaging feature spaces. These characteristics may partly explain why R-MRmr and Relief consistently ranked among the strongest methods across several toxicity endpoints.

In the context of the study, CT image features likely encompass structural characteristics of the head and neck region, including the salivary glands and surrounding tissues. These features could capture aspects such as glandular size, shape, and density, which may influence saliva production and related conditions. However, while CT imaging provides excellent structural detail, its sensitivity to subtle tissue characteristics relevant to saliva production may be limited. Thus, predictive performance using CT image features alone may vary, as observed in the study’s results, where their effectiveness across different conditions and feature selection algorithms fluctuated. $T_{1}$-weighted MRI provides high soft-tissue contrast and may therefore capture variations in parotid tissue composition and heterogeneity that are less apparent on CT. In the present study, $T_{1}$-derived radiomic features frequently showed favorable predictive performance when combined with clinical and dosimetric variables, suggesting that pretreatment tissue characteristics captured by T1-weighted MRI may contain information relevant to radiation-induced salivary dysfunction. However, this interpretation remains hypothesis-generating because the present study was not designed to establish the biological basis of individual radiomic features.

$T_{2}$-weighted MRI images are sensitive to tissue water content and are commonly used to detect abnormalities in soft tissues. $T_{2}$ MRI image features likely provide information about tissue hydration levels and inflammation, which are crucial factors in understanding tumor progress and assessment. Therefore, $T_{2}$ MRI imaging effectiveness in predicting sticky saliva or xerostomia may be influenced by its characteristics, while $T_{1}$-weighted images are better for visualizing normal anatomy and they are useful for side effect monitoring. Therefore, while $T_{2}$ MRI features may offer valuable information, their predictive utility may require careful consideration and further refinement to achieve consistent and reliable results. Notably, combining CT, $T_{1}$-, and $T_{2}$-weighted MRI did not consistently improve predictive performance relative to the strongest individual modality. Multimodal fusion increased the candidate radiomic feature space from 107 features for an individual modality to 321 features, potentially increasing redundancy and model complexity relative to the cohort size. In addition, acquisition-related variability across modalities and institutions may introduce further heterogeneity. Thus, adding imaging modalities does not necessarily improve prediction unless the additional features provide complementary and reproducible information that is effectively retained during feature reduction.

The contrasting performance across input data sets (CT, $T_{1}$, and $T_{2}$ MRI) underscores the importance of feature selection in optimizing predictive models for saliva-related outcomes. While $T_{1}$ MRI data exhibited greater relevance and utility in early prediction tasks, $T_{2}$ MRI data consistently lagged behind in performance metrics. This discrepancy suggests potential differences in the information content and discriminatory power of different imaging modalities in capturing relevant features for saliva-related outcomes. Further research is warranted to elucidate the underlying mechanisms driving these differences and explore potential strategies for enhancing predictive performance, such as novel feature engineering techniques or ensemble modeling approaches.

The present study was designed primarily to benchmark feature-reduction strategies rather than to re-evaluate the incremental predictive contribution of clinical, dosimetric, and radiomic feature domains. The complementary value of combining these information sources has been investigated in our previous radiomics studies. In head and neck cancer, we previously evaluated multimodal models integrating CT and MRI radiomic features with demographic and dosimetric information for prediction of radiation-induced xerostomia and sticky saliva [46,49]. In a separate radiotherapy toxicity application, progressive integration of dosimetric, demographic, clinical, and imaging features demonstrated improved predictive performance compared with models based on more restricted feature domains [22]. Accordingly, the present analysis retained clinical and dosimetric variables within each imaging configuration to provide a consistent modeling framework and focused specifically on comparing the relative performance of different feature-reduction approaches.

An important strength of the present study is the use of a center-independent test cohort. Rather than randomly distributing patients from all three institutions across the training and test sets, model development was restricted to patients from Centers 1 and 2, while Center 3 was completely reserved for independent evaluation. This design provides a more stringent assessment of institutional generalizability because the final models were evaluated on patients acquired at a center that did not contribute to model training or internal validation. This is particularly relevant for radiomics studies, in which differences in scanner hardware and acquisition protocols may influence quantitative feature distributions.

Several limitations should be considered when interpreting these findings. First, although Center 3 was completely withheld from model development and served as an independent institutional test cohort, the external evaluation was limited to a single center comprising 72 patients. Validation in additional independent multicenter cohorts with different patient populations, scanner platforms, and acquisition protocols is therefore warranted. Second, class imbalance, particularly for LateSS, which had the lowest prevalence in the cohort (16%), may have contributed to the lower and more variable predictive performance observed for this endpoint despite the use of SMOTE. The relatively small positive class, together with the high-dimensional multimodal feature space, may also have made identification of stable complementary predictors more challenging. Furthermore, the relatively low prevalence of LateSS limited the precision with which model performance could be characterized, particularly in the independent institutional test cohort. Although ROC-AUC, accuracy, sensitivity, and specificity were evaluated, additional imbalance-sensitive measures, including precision–recall AUC, balanced accuracy, and F1-score, were not reported in the present analysis. Consequently, the performance of the LateSS models should be interpreted cautiously. Future studies with larger numbers of LateSS-positive patients should incorporate these measures and report their uncertainty to provide a more comprehensive evaluation of discrimination and minority-class prediction. Third, the high dimensionality of radiomic data introduces a risk of overfitting even after feature selection. At to this, although parotid contours were independently delineated by two experienced radiation oncologists and finalized by consensus, interobserver radiomic feature reproducibility and the stability of feature selection across resampled training subsets were not formally quantified. Future studies should evaluate feature robustness using reproducibility measures such as the intraclass correlation coefficient (ICC) and assess feature selection stability across repeated resampling procedures. The present study primarily focused on discrimination and comparative performance across feature-reduction strategies. Model calibration and clinical net benefit were not evaluated; therefore, future external validation should include calibration assessment and decision-curve analysis before considering clinical implementation. In addition, the pairwise DeLong comparisons were conducted without adjustment for multiple testing. Given the number of feature-reduction approaches and toxicity endpoints examined, the reported nominal *p*-values may be susceptible to false-positive findings. Accordingly, these comparisons should be considered exploratory and require confirmation in independent cohorts using prespecified comparisons and appropriate multiplicity control. Finally, the present analysis evaluated conventional ML approaches; future studies should investigate whether alternative modeling frameworks and independent prospective validation can further improve toxicity prediction.

## 5. Conclusions

This study demonstrates that feature selection strategy substantially influences the performance of multimodal radiomics models for predicting radiation-induced salivary complications in head and neck cancer. R-MRmr and Relief generally provided the strongest performance, particularly when applied to $T_{1}$-weighted MRI features combined with clinical and dosimetric variables. $T_{1}$-weighted MRI frequently outperformed CT and $T_{2}$-weighted MRI, whereas combining all imaging modalities did not consistently improve discrimination, emphasizing the importance of informative feature reduction rather than simple feature accumulation. Prediction performance varied substantially across toxicity endpoints, with early xerostomia showing particularly strong discrimination and late sticky saliva remaining more challenging. External validation in independent multicenter cohorts is required before clinical implementation.

## Figures and Tables

**Table 1:** Comparing the results of different feature selection algorithms by using Clinical + Dosimetric + Imaging (CT or MRI) features and an SVM classifier to predict EarlySS. Here, since all dosimetric and clinical features are the same for all the categories, we removed it from the table. Values are reported as mean ± SD across repeated evaluations of the complete independent Center 3 test cohort using models generated during repeated model development. Pairwise comparisons of ROC-AUCs between feature selection approaches were performed using the DeLong test on paired patient-level predictions from Center 3, with p<0.05 considered statistically significant.

Algorithm	Features	AUC	ACC	SEN	SPE
**Boruta**	CT	0.65 ± 0.15	0.67 ± 0.11	0.55 ± 0.17	0.78 ± 0.14
	T1	0.80 ± 0.12	0.77 ± 0.15	0.77 ± 0.14	0.57 ± 0.18
	T2	0.61 ± 0.14	0.50 ± 0.12	0.55 ± 0.17	0.44 ± 0.17
	CT + T1 + T2	0.77 ± 0.10	0.65 ± 0.11	0.75 ± 0.13	0.54 ± 0.15
**Kruskal**	CT	0.53 ± 0.15	0.39 ± 0.11	0.45 ± 0.17	0.32 ± 0.16
	T1	0.69 ± 0.14	0.61 ± 0.12	0.56 ± 0.17	0.67 ± 0.16
	T2	0.70 ± 0.17	0.68 ± 0.15	0.68 ± 0.16	0.67 ± 0.17
	CT + T1 + T2	0.66 ± 0.13	0.59 ± 0.12	0.60 ± 0.18	0.54 ± 0.17
**R-MRmr**	CT	0.71 ± 0.13	0.72 ± 0.11	0.78 ± 0.14	0.67 ± 0.17
	T1	**0.85 ± 0.10**	**0.77 ± 0.12**	**0.77 ± 0.16**	**0.75 ± 0.15**
	T2	0.65 ± 0.12	0.66 ± 0.11	0.78 ± 0.14	0.55 ± 0.18
	CT + T1 + T2	0.75 ± 0.14	0.70 ± 0.12	0.69 ± 0.17	0.71 ± 0.17
**Relief**	CT	0.72 ± 0.14	0.66 ± 0.12	0.70 ± 0.18	0.66 ± 0.16
	T1	0.83 ± 0.12	0.72 ± 0.11	0.74 ± 0.17	0.73 ± 0.11
	T2	0.60 ± 0.13	0.56 ± 0.14	0.78 ± 0.14	0.64 ± 0.16
	CT + T1 + T2	0.75 ± 0.14	0.69 ± 0.12	0.65 ± 0.17	0.60 ± 0.17
**RFE**	CT	0.70 ± 0.13	0.61 ± 0.12	0.55 ± 0.18	0.67 ± 0.16
	T1	0.76 ± 0.11	0.74 ± 0.10	0.78 ± 0.15	0.78 ± 0.14
	T2	0.68 ± 0.13	0.72 ± 0.11	0.77 ± 0.14	0.66 ± 0.16
	CT + T1 + T2	0.75 ± 0.14	0.71 ± 0.12	0.69 ± 0.11	0.64 ± 0.15

**Table 2:** Comparing the results of different feature selection algorithms by using Clinical + Dosimetric + Imaging (CT or MRI) features and the SVM classifier to predict EarlyX. Here, since all dosimetric and clinical features are the same for all the categories, we removed it from the table. Values are reported as mean ± SD across repeated evaluations of the complete independent Center 3 test cohort using models generated during repeated model development. Pairwise comparisons of ROC-AUCs between feature selection approaches were performed using the DeLong test on paired patient-level predictions from Center 3, with p<0.05 considered statistically significant.

Algorithm	Features	AUC	ACC	SEN	SPE
**Boruta**	CT	0.76 ± 0.12	0.66 ± 0.11	0.67 ± 0.13	0.66 ± 0.12
	T1	0.91 ± 0.09	0.79 ± 0.10	0.99 ± 0.01	0.77 ± 0.11
	T2	0.65 ± 0.14	0.72 ± 0.12	0.69 ± 0.15	0.71 ± 0.12
	CT + T1 + T2	0.75 ± 0.10	0.71 ± 0.09	0.70 ± 0.11	0.74 ± 0.15
**Kruskal**	CT	0.73 ± 0.15	0.72 ± 0.11	0.67 ± 0.13	0.73 ± 0.12
	T1	0.74 ± 0.13	0.84 ± 0.05	0.79 ± 0.10	0.73 ± 0.10
	T2	0.63 ± 0.13	0.60 ± 0.10	0.69 ± 0.10	0.60 ± 0.10
	CT + T1 + T2	0.74 ± 0.14	0.73 ± 0.12	0.74 ± 0.11	0.64 ± 0.15
**R-MRmr**	CT	0.86 ± 0.14	0.89 ± 0.10	0.75 ± 0.18	0.93 ± 0.10
	T1	**0.96 ± 0.04**	**0.90 ± 0.10**	**0.87 ± 0.10**	**0.93 ± 0.07**
	T2	0.66 ± 0.16	0.57 ± 0.10	0.73 ± 0.13	0.66 ± 0.10
	CT + T1 + T2	0.85 ± 0.11	0.79 ± 0.10	0.84 ± 0.11	0.74 ± 0.15
**Relief**	CT	0.74 ± 0.2	0.61 ± 0.12	0.75 ± 0.15	0.73 ± 0.11
	T1	0.92 ± 0.04	0.83 ± 0.11	0.85 ± 0.15	0.80 ± 0.10
	T2	0.70 ± 0.15	0.67 ± 0.11	0.65 ± 0.17	0.80 ± 0.10
	CT + T1 + T2	0.75 ± 0.18	0.73 ± 0.12	0.79 ± 0.11	0.64 ± 0.15
**RFE**	CT	0.60 ± 0.13	0.67 ± 0.11	0.68 ± 0.21	0.67 ± 0.12
	T1	0.48 ± 0.15	0.44 ± 0.06	0.56 ± 0.24	0.49 ± 0.01
	T2	0.42 ± 0.18	0.52 ± 0.11	0.43 ± 0.13	0.48 ± 0.11
	CT + T1 + T2	0.65 ± 0.15	0.61 ± 0.14	0.71 ± 0.11	0.66 ± 0.15

**Table 3:** Comparing the results of different feature selection algorithms by using Clinical + Dosimetric + Imaging (CT or MRI) features and the SVM classifier to predict LateSS. Here, since all dosimetric and clinical features are the same for all the categories, we removed it from the table. Values are reported as mean ± SD across repeated evaluations of the complete independent Center 3 test cohort using models generated during repeated model development. Pairwise comparisons of ROC-AUCs between feature selection approaches were performed using the DeLong test on paired patient-level predictions from Center 3, with p<0.05 considered statistically significant.

Algorithm	Features	AUC	ACC	SEN	SPE
**Boruta**	CT	0.45 ± 0.18	0.42 ± 0.12	0.47 ± 0.15	0.56 ± 0.14
	T1	0.44 ± 0.15	0.39 ± 0.16	0.59 ± 0.15	0.48 ± 0.13
	T2	0.35 ± 0.12	0.35 ± 0.10	0.63 ± 0.17	0.42 ± 0.15
	CT + T1 + T2	0.45 ± 0.15	0.41 ± 0.14	0.51 ± 0.11	0.46 ± 0.15
**Kruskal**	CT	0.37 ± 0.12	0.34 ± 0.13	0.47 ± 0.17	0.50 ± 0.15
	T1	0.39 ± 0.15	0.33 ± 0.11	0.36 ± 0.15	0.40 ± 0.14
	T2	0.27 ± 0.14	0.24 ± 0.11	0.17 ± 0.36	0.33 ± 0.14
	CT + T1 + T2	0.40 ± 0.15	0.31 ± 0.14	0.41 ± 0.11	0.36 ± 0.15
**R-MRmr**	CT	0.58 ± 0.14	0.39 ± 0.12	0.17 ± 0.17	0.42 ± 0.15
	T1	0.60 ± 0.14	0.5 ± 0.12	0.39 ± 0.11	0.50 ± 0.14
	T2	0.33 ± 0.13	0.28 ± 0.10	0.32 ± 0.2	0.49 ± 0.15
	CT + T1 + T2	0.63 ± 0.15	0.61 ± 0.14	0.67 ± 0.11	0.61 ± 0.15
**Relief**	CT	0.60 ± 0.14	0.59 ± 0.15	0.65 ± 0.16	0.60 ± 0.10
	T1	0.62 ± 0.13	0.60 ± 0.12	0.67 ± 0.12	0.55 ± 0.15
	T2	0.42 ± 0.15	0.43 ± 0.11	0.41 ± 0.22	0.40 ± 0.14
	CT + T1 + T2	**0.65 ± 0.13**	**0.62 ± 0.16**	**0.70 ± 0.15**	**0.65 ± 0.14**
**RFE**	CT	0.38 ± 0.14	0.39 ± 0.12	0.34 ± 0.21	0.42 ± 0.15
	T1	0.26 ± 0.13	0.44 ± 0.12	0.16 ± 0.16	0.58 ± 0.14
	T2	0.39 ± 0.13	0.44 ± 0.13	0.49 ± 0.18	0.67 ± 0.14
	CT + T1 + T2	0.35 ± 0.15	0.31 ± 0.14	0.41 ± 0.11	0.46 ± 0.15

**Table 4:** Comparing the results of different feature selection algorithms by using Clinical + Dosimetric + Imaging (CT or MRI) features and the SVM classifier to predict LateX. Here, since all dosimetric and clinical features are the same for all the categories, we removed it from the table. Values are reported as mean ± SD across repeated evaluations of the complete independent Center 3 test cohort using models generated during repeated model development. Pairwise comparisons of ROC-AUCs between feature-reduction approaches were performed using the DeLong test on paired patient-level predictions from Center 3, with p<0.05 considered statistically significant.

Algorithm	Features	AUC	ACC	SEN	SPE
**Boruta**	CT	0.52 ± 0.14	0.61 ± 0.11	0.64 ± 0.11	0.43 ± 0.16
	T1	0.53 ± 0.12	0.72 ± 0.1	0.78 ± 0.10	0.54 ± 0.14
	T2	0.52 ± 0.15	0.61 ± 0.11	0.75 ± 0.10	0.43 ± 0.13
	CT + T1 + T2	0.62 ± 0.15	0.60 ± 0.12	0.60 ± 0.18	0.64 ± 0.17
**Kruskal**	CT	0.59 ± 0.15	0.55 ± 0.12	0.59 ± 0.13	0.52 ± 0.19
	T1	0.57 ± 0.18	0.54 ± 0.11	0.54 ± 0.14	0.59 ± 0.10
	T2	0.49 ± 0.15	0.55 ± 0.12	0.56 ± 0.12	0.44 ± 0.13
	CT + T1 + T2	0.65 ± 0.11	0.60 ± 0.12	0.67 ± 0.18	0.57 ± 0.17
**R-MRmr**	CT	0.71 ± 0.15	0.69 ± 0.12	0.62 ± 0.14	0.67 ± 0.12
	T1	0.76 ± 0.14	0.68 ± 0.11	0.62 ± 0.13	0.49 ± 0.13
	T2	0.36 ± 0.14	0.67 ± 0.11	0.62 ± 0.10	0.43 ± 0.13
	CT + T1 + T2	0.76 ± 0.13	0.71 ± 0.12	0.73 ± 0.18	0.64 ± 0.17
**Relief**	CT	0.62 ± 0.19	0.55 ± 0.12	0.69 ± 0.13	0.62 ± 0.20
	T1	**0.88 ± 0.11**	**0.81 ± 0.10**	**0.82 ± 0.10**	**0.69 ± 0.14**
	T2	0.49 ± 0.14	0.55 ± 0.12	0.46 ± 0.12	0.33 ± 0.13
	CT + T1 + T2	0.81 ± 0.13	0.79 ± 0.12	0.76 ± 0.18	0.74 ± 0.17
**RFE**	CT	0.65 ± 0.15	0.63 ± 0.11	0.68 ± 0.13	0.58 ± 0.20
	T1	0.74 ± 0.18	0.70 ± 0.12	0.70 ± 0.15	0.64 ± 0.14
	T2	0.56 ± 0.13	0.50 ± 0.11	0.59 ± 0.13	0.53 ± 0.10
	CT + T1 + T2	0.66 ± 0.11	0.59 ± 0.16	0.60 ± 0.15	0.55 ± 0.13

**Table 5:** Summary of the highest AUC achieved by each feature selection approach across the evaluated imaging configurations for the four toxicity endpoints.

Method	EarlySS	EarlyX	LateSS	LateX
Boruta	0.80±0.12	0.91±0.09	0.45±0.18	0.62±0.15
Kruskal–Wallis	0.70±0.17	0.74±0.13	0.40±0.15	0.65±0.11
R-MRmr	0.85±0.10	0.96±0.04	0.63±0.15	0.76±0.14
Relief	0.83±0.12	0.92±0.04	0.65±0.13	0.88±0.11
RFE	0.76±0.11	0.65±0.15	0.39±0.13	0.74±0.18
Forward Selection	0.65±0.10	0.69±0.13	0.42±0.10	0.49±0.11
Backward Elimination	0.60±0.20	0.66±0.12	0.39±0.16	0.56±0.14
PCA	0.61±0.12	0.60±0.16	0.38±0.10	0.54±0.16
LASSO	0.64±0.12	0.65±0.10	0.41±0.12	0.55±0.13

Values represent the highest AUC obtained across the four evaluated imaging configurations (CT, *T*_1_, *T*_2_-weighted MRI, and combined CT + T1 + T2), with clinical and dosimetric features included in each configuration. Complete modality-specific results for Boruta, Kruskal, R-MRmr, Relief, and RFE are presented in Table 1, Table 2, Table 3 and Table 4, while complete results for FS, BE, PCA, and LASSO are provided in Supplementary Tables S7–S10.

## Data Availability

Some part of data used and/or analyzed during the current study are available from the corresponding author on reasonable request and by permission from the university.

## Supplementary Materials

The following supporting information can be downloaded at https://www.mdpi.com/article/10.3390/cancers18193200/s1, Table S1. MRI acquisition protocols used across the three participating centers. Table S2. Demographic, clinical, treatment, and outcome characteristics of the study cohort. Table S3. Mean values for selected dosimetric parameters obtained from the treatment-planning system. Table S4. Classifier performance for prediction of early and late sticky saliva using T1-weighted MRI features. Table S5. Classifier performance for prediction of early and late sticky saliva using CT features. Table S6. Classifier performance for prediction of early and late xerostomia using T1-weighted MRI features. Table S7. Classifier performance for prediction of early and late xerostomia using CT features. Table S8. Hyperparameter search spaces and model settings for the evaluated machine-learning classifiers. Table S9. Performance of additional feature-selection and dimensionality-reduction methods for prediction of early sticky saliva (EarlySS). Table S10. Performance of additional feature-selection and dimensionality-reduction methods for prediction of early xerostomia (EarlyX). Table S11. Performance of additional feature-selection and dimensionality-reduction methods for prediction of late sticky saliva (LateSS). Table S12. Performance of additional feature-selection and dimensionality-reduction methods for prediction of late xerostomia (LateX). Table S13. Implementation details and parameters of the evaluated feature-reduction approaches.
