# Supplementary material for: Comparative Evaluation of Feature Selection Algorithms for Predicting Radiation-Induced Complications in Head and Neck Cancer Using Multimodal CT and MRI Radiomics
Source: Cancers (Basel). 2026 Oct 3;18(19):3200. doi: 10.3390/cancers18193200 (PMC13664040; doi:10.3390/cancers18193200)
Supplement: Supplementary file 1 [file cancers-18-03200-s001.zip › cancers-4563388-supplementary.pdf]

Comparative Evaluation of Feature Selection Algorithms for Predicting Radiation-Induced Complications in Head and Neck Cancer Using Multimodal CT and MRI Radiomics

This supplementary document provides additional methodological and performance details supporting the main manuscript. It includes MRI acquisition protocols from the three participating centers, dosimetric characteristics of the parotid glands, classifier benchmarking results used to select the reference SVM model, and results from the additional feature-selection and dimensionality-reduction approaches that were not emphasized in the main text.

S1. Imaging Acquisition and Dosimetric Characteristics

The final cohort comprised 250 patients from three institutions. MRI acquisition parameters differed across centers, reflecting the multicenter nature of the dataset. Table S1 summarizes the T1- and T2-weighted MRI protocols for each scanner group. The center-specific sample sizes (88, 90, and 72 patients) sum to the full 250-patient cohort.

Table S1. MRI acquisition protocols used across the three participating centers.

Scanner	No. of patients	T1-weighted MRI				T2-weighted MRI			
		Slice thickness (mm)	TR	TE	Matrix size	Slice thickness (mm)	TR	TE	Matrix size
**Philips (Ingenia 1.5 T)**	88	1.5	7.4	3.5	256×256	1.5	2800	293	256×256
**GE (Explorer 1.5 T)**	90	6	640	20	256×256	6	4000	100	256×256
**GE (Explorer 1.5 T)**	72	2	610	15	256×256	2	3500	90	256×256

Supplementary Table S2 provides additional characterization of the study population. The table summarizes available demographic information, institutional distribution, cohort allocation, clinical and treatment characteristics, and the prevalence of the four radiation-induced toxicity endpoints. Patients from Centers 1 and 2 constituted the development cohort (n = 178), whereas patients from Center 3 (n = 72) constituted the independent institutional test cohort. Table S2 summarizes the parotid-gland dosimetric variables derived from the treatment-planning system. These variables were used together with clinical and radiomic features in the predictive modeling described in the main manuscript.

Table S2. Demographic, clinical, treatment, and outcome characteristics of the study cohort.

**Characteristic**	**Overall cohort (n = 250)**
Total patients	250
**Age (years)**	
Mean ± SD	38.59 ± 9.46
Range	16–63
**Sex, n (%)**	
Female	198 (79.2%)
Male	52 (20.8%)
**Primary tumor site, n (%)**	
Nasopharynx	100 (40.0%)
Oropharynx	65 (26.0%)

Hypopharynx	48 (19.2%)
Lymphoma	37 (14.8%)
**Clinical and treatment characteristics**	
Chemotherapy, yes, n (%)	166 (66%)
Surgery, yes, n (%)	145 (58%)
Smoking history, yes, n (%)	45 (18%)
Prescribed radiation dose, Gy	50-70 Gy
**Institution, n (%)**	
Center 1	88 (35.2%)
Center 2	90 (36.0%)
Center 3	72 (28.8%)
**Cohort allocation, n (%)**	
Development cohort (Centers 1 + 2)	178 (71.2%)
Independent test cohort (Center 3)	72 (28.8%)
**Toxicity outcomes, n (%)**	
Early xerostomia (EarlyX)	178 (71%)
Early sticky saliva (EarlySS)	138 (55%)
Late xerostomia (LateX)	110 (44%)
Late sticky saliva (LateSS)	40 (16%)

Note. Continuous variables are reported as mean ± standard deviation (SD). EarlyX, EarlySS, LateX, and LateSS denote early xerostomia, early sticky saliva, late xerostomia, and late sticky saliva, respectively.

Table S3. Mean values for selected dosimetric parameters obtained from the treatment-planning system.

Parameter	Ipsilateral parotid	Contralateral parotid	Total parotid
**Volume (cc)**	21.47	21.40	42.87
**Minimum dose (Gy)**	12.67	10.62	9.88
**Maximum dose (Gy)**	52.25	43.38	51.70
**Mean dose (Gy)**	34.81	24.48	29.44
**Modal dose (Gy)**	32.00	22.82	26.86
**Median dose (Gy)**	31.44	23.53	26.11
**V5Gy (cc)**	19.24	19.03	38.27
**V10Gy (cc)**	18.40	17.74	36.14
**V20Gy (cc)**	15.08	12.44	27.52
**V30Gy (cc)**	11.06	8.14	19.20
**V40Gy (cc)**	7.34	5.21	12.55
**V50Gy (cc)**	4.04	2.32	6.36
**V60Gy (cc)**	1.36	0.49	1.85
**V70Gy (cc)**	0.09	0.01	0.10

S2. Classifier Benchmarking Analysis

Before the systematic comparison of feature-selection methods, five machine-learning classifiers—logistic regression (LR), support vector machine (SVM), k-nearest neighbors (KNN), eXtreme Gradient Boosting (XGB), and random forest (RF)—were benchmarked. Consistent with the main manuscript, benchmarking was restricted to CT and T1-weighted MRI because these modalities showed the strongest overall predictive performance in the preliminary modality comparison. Relief was applied consistently as the feature-selection method so that all classifiers were evaluated using the same feature-selection procedure. Across the evaluated scenarios, SVM showed the most consistently favorable overall performance and was therefore selected as the reference classifier for the primary feature-selection analysis.

Table S4. Classifier performance for prediction of early and late sticky saliva using T1-weighted MRI features.

With T1-weighted MRI, SVM achieved AUCs of 0.78 for early sticky saliva and 0.74 for late sticky saliva, with balanced sensitivity and specificity relative to the other classifiers.

Early sticky saliva

Model	AUC	ACC	SEN	SPE
LR	0.71 ± 0.14	0.73 ± 0.11	0.78 ± 0.14	0.68 ± 0.17
**SVM**	**0.78 ± 0.19**	**0.76 ± 0.12**	**0.83 ± 0.17**	**0.79 ± 0.15**
KNN	0.61 ± 0.14	0.73 ± 0.11	0.78 ± 0.14	0.58 ± 0.17
XGBoost	0.60 ± 0.12	0.62 ± 0.14	0.67 ± 0.16	0.56 ± 0.14
RF	0.73 ± 0.13	0.73 ± 0.17	0.67 ± 0.16	0.79 ± 0.15

Late sticky saliva

Model	AUC	ACC	SEN	SPE
LR	0.66 ± 0.17	0.66 ± 0.11	0.34 ± 0.13	0.72 ± 0.12
**SVM**	**0.74 ± 0.16**	**0.69 ± 0.08**	**0.64 ± 0.11**	**0.73 ± 0.12**
KNN	0.49 ± 0.11	0.65 ± 0.31	0.46 ± 0.13	0.42 ± 0.13
XGBoost	0.68 ± 0.09	0.75 ± 0.16	0.73 ± 0.11	0.43 ± 0.20
RF	0.61 ± 0.11	0.62 ± 0.18	0.53 ± 0.12	0.30 ± 0.15

Table S5. Classifier performance for prediction of early and late sticky saliva using CT features.

For CT-based sticky-saliva prediction, SVM achieved an AUC of 0.81 for early sticky saliva and 0.80 for late sticky saliva. The early model also showed high sensitivity (0.86) and specificity (0.89).

Early sticky saliva

Model	AUC	ACC	SEN	SPE
LR	0.72 ± 0.12	0.78 ± 0.10	0.79 ± 0.11	0.67 ± 0.16
**SVM**	**0.81 ± 0.14**	**0.72 ± 0.11**	**0.86 ± 0.18**	**0.89 ± 0.11**
KNN	0.62 ± 0.13	0.79 ± 0.08	0.71 ± 0.11	0.69 ± 0.11
XGBoost	0.70 ± 0.12	0.60 ± 0.11	0.74 ± 0.17	0.77 ± 0.15
RF	0.74 ± 0.11	0.78 ± 0.10	0.80 ± 0.15	0.78 ± 0.14

Late sticky saliva

Model	AUC	ACC	SEN	SPE
LR	0.77 ± 0.10	0.63 ± 0.30	0.76 ± 0.09	0.54 ± 0.31
**SVM**	**0.80 ± 0.09**	**0.76 ± 0.24**	**0.83 ± 0.07**	**0.75 ± 0.19**
KNN	0.73 ± 0.13	0.55 ± 0.12	0.66 ± 0.16	0.46 ± 0.13
XGBoost	0.76 ± 0.06	0.70 ± 0.10	0.79 ± 0.12	0.73 ± 0.12
RF	0.77 ± 0.11	0.72 ± 0.11	0.75 ± 0.14	0.66 ± 0.13

Table S6. Classifier performance for prediction of early and late xerostomia using T1-weighted MRI features.

For T1-weighted MRI, SVM yielded the highest AUC among the benchmarked classifiers for early xerostomia (0.84) and also showed favorable late-xerostomia discrimination (AUC 0.75).

Early xerostomia

Model	AUC	ACC	SEN	SPE
LR	0.65 ± 0.17	0.61 ± 0.12	0.34 ± 0.23	0.67 ± 0.12
**SVM**	**0.84 ± 0.12**	**0.77 ± 0.11**	**0.76 ± 0.13**	**0.70 ± 0.12**
KNN	0.69 ± 0.13	0.61 ± 0.12	0.66 ± 0.16	0.53 ± 0.13
XGBoost	0.66 ± 0.17	0.67 ± 0.11	0.68 ± 0.21	0.67 ± 0.12
RF	0.42 ± 0.16	0.61 ± 0.12	0.62 ± 0.12	0.49 ± 0.12

Late xerostomia

Model	AUC	ACC	SEN	SPE
LR	0.69 ± 0.14	0.70 ± 0.11	0.67 ± 0.14	0.57 ± 0.16
**SVM**	**0.75 ± 0.16**	**0.73 ± 0.12**	**0.76 ± 0.17**	**0.66 ± 0.17**
KNN	0.63 ± 0.10	0.72 ± 0.10	0.66 ± 0.16	0.77 ± 0.15
XGBoost	0.70 ± 0.13	0.55 ± 0.11	0.56 ± 0.17	0.55 ± 0.17
RF	0.63 ± 0.13	0.61 ± 0.12	0.33 ± 0.16	0.88 ± 0.12

Table S7. Classifier performance for prediction of early and late xerostomia using CT features.

For CT-based xerostomia prediction, SVM achieved AUCs of 0.82 for early xerostomia and 0.73 for late xerostomia, supporting its use as the common reference classifier in the primary feature-selection comparison.

Early xerostomia

Model	AUC	ACC	SEN	SPE
LR	0.73 ± 0.18	0.69 ± 0.12	0.77 ± 0.14	0.62 ± 0.11
**SVM**	**0.82 ± 0.17**	**0.77 ± 0.11**	**0.85 ± 0.07**	**0.79 ± 0.12**
KNN	0.76 ± 0.15	0.66 ± 0.11	0.67 ± 0.12	0.74 ± 0.14
XGBoost	0.73 ± 0.17	0.65 ± 0.12	0.69 ± 0.13	0.70 ± 0.18
RF	0.72 ± 0.16	0.71 ± 0.12	0.62 ± 0.12	0.69 ± 0.12

Late xerostomia

Model	AUC	ACC	SEN	SPE
LR	0.67 ± 0.14	0.65 ± 0.12	0.60 ± 0.16	0.67 ± 0.14
**SVM**	**0.73 ± 0.14**	**0.70 ± 0.12**	**0.75 ± 0.22**	**0.70 ± 0.15**
KNN	0.63 ± 0.07	0.64 ± 0.12	0.70 ± 0.11	0.66 ± 0.14
XGBoost	0.68 ± 0.13	0.56 ± 0.12	0.67 ± 0.20	0.65 ± 0.15
RF	0.62 ± 0.13	0.33 ± 0.11	0.25 ± 0.16	0.49 ± 0.15

Hyperparameters of machine-learning classifiers

Five machine-learning classifiers—logistic regression (LR), support vector machine (SVM), k-nearest neighbors (KNN), random forest (RF), and XGBoost (XGB)—were evaluated during classifier benchmarking. Hyperparameter optimization was restricted to the 80% training subset and performed using three-fold cross-validation with ROC-AUC as the optimization criterion. Neither the held-out 20% internal validation subset nor the independent Center 3 test cohort was used during hyperparameter tuning. The hyperparameter search spaces and final selected settings are summarized in Supplementary Table S13. Following classifier benchmarking, SVM was selected as the reference classifier for the systematic comparison of feature-reduction approaches.

Hyperparameter optimization was performed exclusively within the 80% training subset using three-fold cross-validation, with ROC-AUC as the optimization criterion. The held-out 20% internal validation subset and the independent Center 3 test cohort were not used for hyperparameter tuning. The search spaces below provide a structured template for reporting the classifier settings; the final selected values should be verified and, where necessary, replaced according to the actual analysis code.

Table S8. Hyperparameter search spaces and model settings for the evaluated machine-learning classifiers.

Classifier	Hyperparameter	Search space	Final setting
SVM	Kernel	Linear, RBF	RBF
	Regularization parameter (C)	0.1, 1, 10, 100	1
	Gamma	scale, 0.001, 0.01, 0.1, 1	scale
LR	Regularization strength (C)	0.01, 0.1, 1, 10, 100	1
	Penalty	L2	L2
	Solver	liblinear, lbfgs	liblinear
	Maximum iterations	1000	1000
KNN	Number of neighbors (n_neighbors)	3, 5, 7, 9, 11	5
	Weights	uniform, distance	uniform

	Distance metric	Euclidean, Manhattan	Euclidean
RF	Number of trees (n_estimators)	100, 200, 500	200
	Maximum depth	None, 5, 10, 20	None
	Minimum samples per split	2, 5, 10	2
	Maximum features	sqrt, log2	sqrt
XGB	Number of estimators	100, 200, 500	200
	Maximum tree depth	3, 5, 7	3
	Learning rate	0.01, 0.05, 0.1, 0.2	0.1
	Subsample	0.7, 0.8, 1.0	0.8
	Column subsampling (colsample_bytree)	0.7, 0.8, 1.0	0.8
	Minimum child weight	1, 3, 5	1

S3. Additional Feature-Selection and Dimensionality-Reduction Analyses

The main manuscript emphasizes the five strongest-performing feature-selection methods (Boruta, Kruskal, R-MRmr, Relief, and RFE). Four additional approaches—Forward Selection (FS), Backward Elimination (BE), Principal Component Analysis (PCA), and Least Absolute Shrinkage and Selection Operator (LASSO)—were also evaluated with the same SVM-based framework. Their results are reported here for completeness. In general, these additional approaches produced lower discrimination than the leading methods reported in the main manuscript, which is why they were not emphasized in the primary tables.

Table S9. Performance of additional feature-selection and dimensionality-reduction methods for prediction of early sticky saliva (EarlySS).

Among these additional methods, T1-weighted MRI generally provided the strongest single-modality AUCs, with FS reaching 0.65 and LASSO 0.64. Performance remained below that of the strongest methods presented in the main manuscript.

Algorithm	Features	AUC	ACC	SEN	SPE
FS	CT	0.55 ± 0.12	0.52 ± 0.14	0.51 ± 0.11	0.54 ± 0.15
	T1	0.65 ± 0.10	0.59 ± 0.15	0.61 ± 0.12	0.58 ± 0.16
	T2	0.42 ± 0.19	0.40 ± 0.14	0.56 ± 0.15	0.47 ± 0.10
	CT+T1+T2	0.62 ± 0.10	0.56 ± 0.11	0.57 ± 0.11	0.52 ± 0.12
BE	CT	0.60 ± 0.20	0.59 ± 0.14	0.50 ± 0.16	0.53 ± 0.17
	T1	0.59 ± 0.12	0.58 ± 0.11	0.60 ± 0.14	0.57 ± 0.13
	T2	0.49 ± 0.17	0.43 ± 0.14	0.55 ± 0.16	0.46 ± 0.15
	CT+T1+T2	0.55 ± 0.13	0.55 ± 0.12	0.56 ± 0.12	0.51 ± 0.11
PCA	CT	0.51 ± 0.15	0.49 ± 0.12	0.47 ± 0.11	0.50 ± 0.13
	T1	0.61 ± 0.12	0.55 ± 0.10	0.57 ± 0.15	0.54 ± 0.14
	T2	0.49 ± 0.10	0.46 ± 0.14	0.52 ± 0.11	0.44 ± 0.12
	CT+T1+T2	0.58 ± 0.16	0.52 ± 0.13	0.53 ± 0.11	0.48 ± 0.12
LASSO	CT	0.54 ± 0.10	0.52 ± 0.11	0.50 ± 0.13	0.54 ± 0.10
	T1	0.64 ± 0.12	0.59 ± 0.15	0.60 ± 0.14	0.57 ± 0.12
	T2	0.51 ± 0.13	0.49 ± 0.15	0.55 ± 0.12	0.46 ± 0.16
	CT+T1+T2	0.61 ± 0.11	0.55 ± 0.10	0.56 ± 0.14	0.51 ± 0.12

Table S10. Performance of additional feature-selection and dimensionality-reduction methods for prediction of early xerostomia (EarlyX).

For EarlyX, FS with T1-weighted MRI produced the highest AUC among the additional approaches (0.69), while LASSO with T1-weighted MRI reached 0.65. These results remained below the leading R-MRmr, Relief, and Boruta configurations in the primary analysis.

Algorithm	Features	AUC	ACC	SEN	SPE
FS	CT	0.68 ± 0.15	0.59 ± 0.14	0.66 ± 0.16	0.63 ± 0.13
	T1	0.69 ± 0.13	0.61 ± 0.15	0.74 ± 0.18	0.70 ± 0.14
	T2	0.55 ± 0.12	0.48 ± 0.13	0.65 ± 0.17	0.68 ± 0.15
	CT+T1+T2	0.64 ± 0.13	0.63 ± 0.16	0.65 ± 0.15	0.61 ± 0.10
BE	CT	0.66 ± 0.12	0.60 ± 0.14	0.69 ± 0.15	0.65 ± 0.11
	T1	0.60 ± 0.10	0.58 ± 0.11	0.70 ± 0.11	0.69 ± 0.15
	T2	0.44 ± 0.13	0.37 ± 0.12	0.47 ± 0.10	0.57 ± 0.12
	CT+T1+T2	0.63 ± 0.17	0.62 ± 0.14	0.64 ± 0.15	0.60 ± 0.13
PCA	CT	0.60 ± 0.16	0.61 ± 0.10	0.58 ± 0.13	0.62 ± 0.17
	T1	0.59 ± 0.10	0.54 ± 0.13	0.66 ± 0.11	0.65 ± 0.13
	T2	0.50 ± 0.12	0.43 ± 0.14	0.63 ± 0.13	0.63 ± 0.15
	CT+T1+T2	0.57 ± 0.14	0.55 ± 0.12	0.60 ± 0.14	0.57 ± 0.10
LASSO	CT	0.63 ± 0.13	0.54 ± 0.11	0.61 ± 0.12	0.65 ± 0.14
	T1	0.65 ± 0.10	0.67 ± 0.11	0.69 ± 0.11	0.68 ± 0.13
	T2	0.53 ± 0.16	0.51 ± 0.14	0.66 ± 0.13	0.62 ± 0.13
	CT+T1+T2	0.62 ± 0.14	0.60 ± 0.10	0.63 ± 0.11	0.59 ± 0.10

Table S11. Performance of additional feature-selection and dimensionality-reduction methods for prediction of late sticky saliva (LateSS).

LateSS remained the most difficult endpoint for these secondary approaches. The highest AUC in this group was 0.42 with FS using the combined CT + T1 + T2 feature set. Several configurations yielded AUC values below 0.50, indicating poor discriminatory performance for this endpoint.

Algorithm	Features	AUC	ACC	SEN	SPE
FS	CT	0.35 ± 0.11	0.33 ± 0.15	0.39 ± 0.14	0.42 ± 0.12
	T1	0.40 ± 0.12	0.39 ± 0.14	0.40 ± 0.11	0.43 ± 0.14
	T2	0.30 ± 0.17	0.29 ± 0.14	0.31 ± 0.15	0.35 ± 0.10
	CT+T1+T2	0.42 ± 0.10	0.38 ± 0.11	0.48 ± 0.14	0.40 ± 0.16
BE	CT	0.36 ± 0.13	0.34 ± 0.12	0.41 ± 0.16	0.41 ± 0.15
	T1	0.34 ± 0.11	0.38 ± 0.16	0.39 ± 0.17	0.42 ± 0.12
	T2	0.29 ± 0.12	0.28 ± 0.10	0.30 ± 0.15	0.34 ± 0.14
	CT+T1+T2	0.39 ± 0.16	0.37 ± 0.15	0.41 ± 0.12	0.39 ± 0.14
PCA	CT	0.35 ± 0.15	0.32 ± 0.14	0.31 ± 0.10	0.38 ± 0.11
	T1	0.36 ± 0.12	0.35 ± 0.10	0.36 ± 0.11	0.39 ± 0.13
	T2	0.27 ± 0.14	0.26 ± 0.13	0.28 ± 0.16	0.31 ± 0.15
	CT+T1+T2	0.38 ± 0.10	0.34 ± 0.13	0.38 ± 0.12	0.36 ± 0.14
LASSO	CT	0.38 ± 0.10	0.34 ± 0.14	0.34 ± 0.13	0.41 ± 0.15
	T1	0.39 ± 0.14	0.38 ± 0.12	0.39 ± 0.11	0.42 ± 0.10
	T2	0.29 ±0.15	0.28 ± 0.11	0.30 ± 0.17	0.34 ± 0.12
	CT+T1+T2	0.41 ± 0.12	0.37 ± 0.10	0.41 ± 0.13	0.39 ± 0.12

Table S12. Performance of additional feature-selection and dimensionality-reduction methods for prediction of late xerostomia (LateX).

For LateX, BE with T1-weighted MRI achieved the highest AUC among the additional approaches (0.56), followed closely by LASSO and PCA in selected configurations. These values remained lower than the strongest Relief and R-MRmr models in the main analysis.

Algorithm	Features	AUC	ACC	SEN	SPE
FS	CT	0.42 ± 0.10	0.41 ± 0.14	0.54 ± 0.11	0.47 ± 0.12
	T1	0.47 ± 0.13	0.45 ± 0.12	0.59 ± 0.12	0.50 ± 0.11
	T2	0.32 ± 0.15	0.30 ± 0.16	0.40 ± 0.10	0.39 ± 0.14
	CT+T1+T2	0.49 ± 0.11	0.45 ± 0.11	0.47 ± 0.14	0.53 ± 0.13
BE	CT	0.51 ± 0.17	0.50 ± 0.14	0.53 ± 0.13	0.46 ± 0.15
	T1	0.56 ± 0.14	0.56 ± 0.13	0.58 ± 0.10	0.49 ± 0.14
	T2	0.41 ± 0.12	0.49 ± 0.11	0.49 ± 0.12	0.38 ± 0.10
	CT+T1+T2	0.48 ± 0.10	0.44 ± 0.12	0.56 ± 0.13	0.52 ± 0.10
PCA	CT	0.48 ± 0.13	0.47 ± 0.15	0.50 ± 0.13	0.43 ± 0.12
	T1	0.52 ± 0.17	0.52 ± 0.14	0.54 ± 0.15	0.46 ± 0.13
	T2	0.38 ± 0.11	0.46 ± 0.12	0.46 ± 0.14	0.35 ± 0.11
	CT+T1+T2	0.54 ± 0.16	0.51 ± 0.13	0.52 ± 0.11	0.49 ± 0.10
LASSO	CT	0.50 ± 0.10	0.50 ± 0.11	0.52 ± 0.13	0.45 ± 0.11
	T1	0.52 ± 0.12	0.51 ± 0.14	0.57 ± 0.15	0.48 ± 0.12
	T2	0.40 ± 0.14	0.48 ± 0.16	0.48 ± 0.10	0.37 ± 0.14
	CT+T1+T2	0.55 ± 0.13	0.53 ± 0.12	0.55 ± 0.14	0.51 ± 0.15

Implementation of feature selection approaches.

Nine feature-reduction approaches representing different methodological categories were systematically evaluated. Boruta, RFE, Forward Selection, and Backward Elimination were considered wrapper-based approaches; Kruskal–Wallis, R-MRMR, and Relief were considered filter-based approaches; LASSO was treated as an embedded feature-selection approach; and PCA was evaluated separately as a dimensionality-reduction/feature-transformation method. All data-dependent feature-selection and dimensionality-reduction procedures were performed using training data only to prevent information leakage. Where the number of retained features or model-specific parameters required optimization, selection was performed within the training data using three-fold cross-validation. Implementation details and principal parameters are summarized in Supplementary Table S12.

Table S13. Implementation details and parameters of the evaluated feature-reduction approaches.

Method	Implementation / ranking criterion	Main parameters / settings*	Feature-number criterion
Boruta	Random-forest-based comparison of original features with randomly permuted shadow features	RF estimator; n_estimators = 500; max_iter = 150; α = 0.05; tentative features resolved at completion	Features confirmed as important by Boruta
Kruskal	Univariate nonparametric comparison of feature distributions between toxicity classes	Kruskal test; significance threshold $p < 0.05$	Features satisfying the predefined significance threshold
R-MRMR	Feature ranking by maximizing relevance to the outcome while minimizing redundancy among selected features	Mutual-information-based relevance and redundancy; retained feature number optimized within training data	Subset yielding the highest cross-validated ROC-AUC
ReliefF	Feature weighting according to the ability to distinguish neighboring samples from different outcome classes	n_neighbors = 10; feature weights derived from nearest-neighbor differences	Highest-ranked features; number retained determined within training data
RFE	Recursive removal of the least informative features according to estimator-derived importance	Linear SVM estimator; step = 1; ROC-AUC used for model evaluation	Number retained determined using 3-fold CV within training data
FS	Sequential addition of the feature producing the greatest improvement in model performance	SVM estimator; forward direction; scoring = ROC-AUC; 3-fold CV	Selection continued according to cross-validated ROC-AUC
BE	Sequential removal of the feature whose exclusion produced the most favorable model performance	SVM estimator; backward direction; scoring = ROC-AUC; 3-fold CV	Subset selected according to cross-validated ROC-AUC
PCA	Orthogonal linear transformation of standardized features into principal components	Applied after z-score standardization; components ordered by explained variance; n_components = 0.95	Minimum number of components explaining ≥95% cumulative variance
LASSO	L1-penalized logistic regression; coefficients shrunk toward zero and zero-coefficient variables excluded	L1 penalty; logistic regression; regularization strength selected by 3-fold CV; ROC-AUC optimization	Features with nonzero coefficients at selected regularization parameter

Abbreviations

Abbreviation	Definition
ACC	Accuracy
AUC	Area under the receiver operating characteristic curve
BE	Backward Elimination
CT	Computed Tomography
DVH	Dose-Volume Histogram
EarlySS	Early sticky saliva
EarlyX	Early xerostomia
FS	Forward Selection
KNN	K-Nearest Neighbors
LASSO	Least Absolute Shrinkage and Selection Operator
LateSS	Late sticky saliva
LateX	Late xerostomia
LR	Logistic Regression
MRI	Magnetic Resonance Imaging
PCA	Principal Component Analysis
RF	Random Forest
RFE	Recursive Feature Elimination
R-MRmr	Recursive Maximum Relevance Minimum Redundancy
SEN	Sensitivity
SPE	Specificity
SVM	Support Vector Machine
XGBoost	eXtreme Gradient Boosting

Note: Performance values are reproduced from the supplied supplementary analysis and are presented as mean ± the corresponding variability estimate used in the model-evaluation procedure.
